# Knowledge, attitudes, behaviors, and information needs of women vaccinated with the HPV vaccine regarding cervical cancer prevention: a cross-sectional study

**DOI:** 10.3389/fpubh.2025.1493589

**Published:** 2025-02-13

**Authors:** Xuan Zhou, Miaomiao Wu, Yuling Zhou, Fang Su, Yiqing He, Jinxia Ding, Lunfang Xie

**Affiliations:** ^1^School of Nursing, Anhui Medical University, Hefei, Anhui, China; ^2^Department of Medical Oncology, First Affiliated Hospital of Anhui Medical University, Hefei, Anhui, China

**Keywords:** cervical cancer, HPV vaccine, screening, knowledge, attitude, behavior, information needs

## Abstract

**Background:**

Cervical cancer poses a serious threat to women’s health globally, especially in China. HPV vaccination and screening are crucial prevention and control measures. However, the screening coverage among Chinese women remains low, and there is a need to better understand the knowledge, attitudes, behaviors, and information needs of Chinese HPV-vaccinated women regarding cervical cancer prevention to optimize prevention and control strategies.

**Objectives:**

To explore the knowledge, attitudes, behaviors, and information needs of women vaccinated with the HPV vaccine regarding cervical cancer prevention.

**Methods:**

This cross-sectional study was conducted using a convenience sampling method from October 1 to December 30, 2023. A questionnaire survey was administered to 439 women vaccinated with the HPV vaccine at the Shu Shan District Community Health Service Center in Hefei, Anhui Province. The survey tool was self-designed. Data were analyzed using descriptive statistics, chi-square tests, and binary logistic regression.

**Results:**

The average age of the 439 participants was 27.82 ± 6.42 years. The average cervical cancer prevention knowledge score was 35.01 ± 5.76. 434 (98.9%) women held a positive attitude towards cervical cancer screening, and 320 (72.9%) women had undergone cervical cancer screening after receiving the HPV vaccine. Educational levels such as college (OR = 2.995, 95%CI: 1.233–7.279, *p* = 0.015), bachelor’s degree (OR = 3.694, 95%CI: 1.718–7.943, *p* = 0.001), and postgraduate and above (OR = 4.826, 95%CI: 2.176–10.707, *p* < 0.001), as well as occupation as medical workers (OR = 4.660, 95%CI: 2.292–9.474, *p* < 0.001), were associated with higher knowledge of prevention and treatment scores. Individuals aged 26–35 years (OR = 7.431, 95%CI: 2.856–19.331, *p* < 0.001), 36–45 years (OR = 11.466, 95%CI: 2.279–57.694, *p* = 0.003), married individuals (OR = 4.307, 95%CI: 1.455–12.750, *p* = 0.008), and participants who had received health education related to cervical cancer prevention (OR = 2.125, 95%CI: 1.169–3.863, *p* = 0.013) and possessed good knowledge of prevention (OR = 16.770, 95%CI: 8.667–32.451, *p* < 0.001) were more inclined to undergo cervical cancer screening. Among the 254 participants who had received health education, 34.2% still had unmet information needs regarding cervical cancer prevention, and 29.5% hoped to receive health education services from professionals.

**Conclusion:**

Chinese HPV-vaccinated women have a good understanding of cervical cancer prevention and a positive attitude and behavior towards cervical cancer screening. However, their knowledge of cervical cancer screening is not sufficient, and their information needs have not been fully met.

## Introduction

Cervical cancer is one of the three most common malignancies in women worldwide (1). In low- and middle-income countries, the incidence and mortality of cervical cancer far exceed those in developed countries, posing a severe threat to women’s health. China has the highest number of cervical cancer cases globally (2), with approximately 110,000 new cases and 59,000 deaths annually, accounting for 18.2 and 17.3% of the global incidence and mortality rates, respectively, and showing a trend of younger age at onset (3). This dire situation underscores the urgency of strengthening cervical cancer prevention and control efforts. The cause of cervical cancer is clear, and it is mainly caused by high-risk human neoplastic virus (human papilloma virus HPV), which can be prevented and controlled. It takes years or even decades for women to progress from high-risk HPV infection to cervical intraepithelial neoplasia and eventually to cervical cancer (4). Currently, cervical cancer prevention and control focuses on primary and secondary prevention. Primary prevention involves HPV vaccination in women of appropriate age, while secondary prevention includes cervical cancer screening and early diagnosis and treatment for eligible women.

Many developed countries have demonstrated that long-term and effective cervical cancer screening and HPV vaccination programs can significantly reduce the incidence and mortality of cervical cancer. Since the introduction of HPV vaccines in the United States, the vaccines have effectively reduced HPV infection rates, playing an increasingly significant role in cancer prevention and enhancing herd immunity (5). Data from Sweden also show a substantial reduction in the risk of invasive cervical cancer associated with HPV vaccination (6). In addition, after implementing cervical cancer screening programs, cervical cancer mortality rates in different regions of Europe have declined by 51–92% (7). However, due to a lack of understanding of cervical cancer screening and HPV vaccination programs, adoption rates of these programs are low in many developing countries (8). In Pakistan, alarmingly, up to 70% of women are diagnosed with advanced-stage malignancies, leading to high mortality rates in the country (9). Similarly, in Saudi Arabia, over 40% of cervical cancer cases are detected at late stages (III and IV), making treatment more challenging and prognosis poorer (10). With the gradual introduction of HPV vaccines in China, the HPV vaccination rate among of appropriate age in China has increased significantly (11). However, it is worth noting that the long-term efficacy of the vaccine has not been fully established, and the vaccine cannot eliminate existing HPV infections, genital warts, or precursor lesions, nor can it prevent all HPV subtypes that can cause cervical cancer. In China, only 30.0% of urban women and 22.6% of rural women aged 20–64 have undergone cervical cancer screening, indicating a significant deficiency in cervical cancer screening coverage in China (12). The American College of Obstetricians and Gynaecologists 2016 guidelines for cervical cancer screening and prevention clearly state that women who have received HPV vaccines should still undergo regular screening according to the guidelines for women who have not been vaccinated (13). The World Health Organization (WHO) points out that a combination of Pap smear testing and HPV vaccination is an effective measure to eradicate cervical cancer as a global public health issue (14). Therefore, women who have received HPV vaccines should not let their guard down and need to undergo regular cervical cancer screening. How to do well in primary and secondary prevention of cervical cancer is the focus of cervical cancer prevention work, which largely depends on the public’s awareness of cervical cancer prevention and control knowledge, attitudes towards screening, and screening behaviors. Studies by Shamaun et al. (15) on female patients in gynecological outpatient clinics in Pakistan revealed that only a few patients understood cervical cancer, cervical screening, and HPV vaccines, and most lacked knowledge of risk factors. Although some patients were willing to participate in screening, few did so. A cross-sectional study by Heena et al. (16) on cervical cancer knowledge among female healthcare professionals, primarily nurses, found that while most respondents recognized the effectiveness of Pap smear testing, only a minority had undergone the test. A study by Alshammiri (17) on female high school teachers in Hail City showed that only a few teachers had a deep understanding of cervical cancer, and few underwent screening. Although some teachers believed in the usefulness of screening, overall knowledge levels and screening practices were inadequate. Survey data indicate (18) that the incidence of cervical cancer among women of reproductive age in China remains high, which may be related to the low prevalence of regular cervical cancer screening and insufficient knowledge of prevention among women. However, there are no reports on cervical cancer prevention knowledge, attitudes, behaviors, and information needs among women who have received HPV vaccines in China.

Therefore, this study surveyed cervical cancer prevention knowledge, attitudes, behaviors, and information needs of women who have been vaccinated with the HPV vaccine to comprehensively understand the prevention literacy and health education needs of vaccinated women and provide more targeted suggestions for optimizing prevention and control strategies.

## Materials and methods

### Study subjects

Offline participants were selected through convenient sampling at the Shushan District Community Health Service Center in Hefei City (the community health service center serves a population of approximately 89,000 people, providing public health services and essential medical services, and regularly providing free examinations and condition monitoring for the older adult, women, children, and patients with chronic diseases). Online participants were recruited through recommendations from offline participants. Inclusion criteria: Females who have received at least one dose of HPV vaccine; Exclusion criteria: Females with mental illness or language barriers.

### Sample size

Based on the formula *n* = *μ_α_* ^2^*P*(1-*Ρ*)/*δ*^2^, the pre-survey showed that the good knowledge rate among women who have received the HPV vaccine was *p* = 79%, with a significance level of *α* = 0.05 and an allowable error of *δ* = 4%. Considering an additional 5% invalid samples, the required sample size was 420 cases. Four hundred and sixty-four people participated in the survey, and 439 valid questionnaires were obtained.

### Questionnaire

Based on relevant literature (19–23), after thorough discussion within the research team, we designed the “*Questionnaire on Cervical Cancer Prevention Knowledge, Attitudes, and Behaviors Among Women Who Have Received HPV Vaccine*.” To ensure the validity and comprehensibility of the questionnaire, before the formal survey, we invited 50 women who had received the HPV vaccine to participate in an online pre-survey. Based on their feedback, we made corresponding adjustments to the questionnaire. Specifically, we added the dimension of information needs to better understand the participants’ information acquisition needs and preferences related to cervical cancer prevention.

The final questionnaire was divided into two parts, with 53 items. The first part was about sociodemographic characteristics, including 11 items such as age and marital status; the second part, the main body of the questionnaire, comprised 42 items, including 22 items on cervical cancer prevention knowledge, mainly covering HPV and cervical cancer knowledge as well as cervical cancer screening Knowledge, 5 items related to attitudes towards cervical cancer prevention, 3 items related to the behaviors associated to cervical cancer prevention, and 12 items on information needs for cervical cancer prevention knowledge. Each part adopted a combination of single-choice and multiple-choice questions. The cervical cancer prevention knowledge section included 16 single-choice and 6 multiple-choice questions. For single-choice questions, a correct answer was scored as 1 point, and an incorrect answer as 0 points. A correct answer was scored as 1 point for multiple-choice questions, and wrong answers were not penalized. The total score for this section was 47 points. Participants with a total knowledge score of 33 points or above (70%) were considered to have good knowledge levels. The higher the score, the higher the cognition, attitude, and behavior level. The specific content of the questionnaire is shown in Supplementary material 1.

### Data collection methods

Wenjuanxing (an online survey platform) and paper-based questionnaires were used to collect data by two graduate nursing students who have already received training. If the offline participants were equipped with internet access and preferred to fill out the electronic questionnaire, they were asked to scan the QR (Quick Response) code of the electronic questionnaire prepared in advance. If they did not have access to the internet or preferred to fill out the paper-based questionnaires, the paper version was used for the survey. After the survey was completed, the investigator asked the respondents with mobile phone accessing the internet whether they had any female acquaintance who met the recruitment conditions. If so, the investigator sent the QR code image of the questionnaire, the survey instructions (including the investigator’s contact information) and the privacy protection statement to the referrer, asking her to forward them to the referred person. The whole procedure was supervised by the investigators. In addition, for online survey, the first question of the questionnaire was “Have you been vaccinated against HPV?.” If the participant selected “No,” the survey was automatically closed. The same IP address could only be accessed once. The questionnaires were regarded as invalid, if their filling time less than 150 s, or they had obvious logical errors or the same options.

### Data analysis

A database was established using EpiData 3.1 for paper-based questionnaires, and a double parallel entry was adopted. SPSS 24.0 was used for statistical analysis of the data. Normally distributed data were described using means and standard deviations, while non-normally distributed data were described using medians and interquartile ranges. Count data were described using proportions and rates. Univariate and multivariate analysis methods were used sequentially to identify the main factors influencing cervical cancer prevention knowledge and screening behaviors among the study participants.

## Results

### General information of participants

A total of 464 individuals participated in the survey, with 185 questionnaires collected online, of which 181 were valid, resulting in an effective response rate of 97.84%. Offline, 279 questionnaires were collected, with 258 being valid, yielding an effective response rate of 92.47%. Combining online and offline, 439 valid questionnaires were obtained, with an overall effective response rate of 94.61%. The average age of the 439 participants was 27.82 ± 6.42 years. Among the participants, 45.8% held a bachelor’s degree, and 29.8% had a master’s degree or higher. Additionally, 62.0% of the respondents were unmarried. See Table 1 for details.

**Table 1:** General information of survey participants (*n* = 439).

Category	Classification	Number	Percentage (%)
Age	14 ~ 25	222	50.6
	26 ~ 35	148	33.7
	36 ~ 45	69	15.7
Education level	High School/Technical School and Below	36	8.2
	Junior College	71	16.2
	Bachelor’s Degree	201	45.8
	Master’s Degree and Above	131	29.8
Marital status	Unmarried	272	62.0
	Married	162	36.9
	Divorced or Widowed	5	1.1
Occupation	Student	145	33.0
	Medical Worker	94	21.4
	Educator	37	8.4
	Self-employed or Freelancer	77	17.5
	Enterprise/Institution Worker	86	19.6
Residence	Rural Area	87	19.8
	City	256	58.3
	Township	40	9.1
	County Town	56	12.8
Monthly family income (Yuan)	<3,000	23	5.2
	3,001 ~ 5,000	64	14.6
	5,001 ~ 10,000	152	34.6
	>10,000	200	45.6

### Additional information on participants

Regarding vaccination status, 305 individuals (69.5%) had received the nine-valent HPV vaccine, while 48 (10.9%) had received the bivalent HPV vaccine, and 86 (19.6%) had received the quadrivalent HPV vaccine. Among the participants, 98 (22.3%) had undergone a gynecological examination within the past 3 months, and 6 (1.4%) reported having a family history of cervical cancer.

### Health education on cervical cancer prevention received by participants

57.8% of the participants had previously received health education about cervical cancer prevention. The most common venues for such education were community health centers (35.0%), campuses (26.1%), and online virtual platforms (19.8%).

### Knowledge of cervical cancer prevention

The participants’ average cervical cancer prevention knowledge assessment score was 35.01 ± 5.76 points. Three hundred one participants (68.6%) demonstrated good knowledge, while 138 (31.4%) participants had poor knowledge. Table 2 shows the five items with the lowest correct response rates.

**Table 2:** Five topics with low rates of correct cervical cancer prevention knowledge in women who have been vaccinated against HPV.

Topic	Options	The accuracy of each option in the multiple-choice question (%)	Total correctness (%)
K1. HPV infection typically does not present with noticeable symptoms.			44.2
K2. The purpose of cervical cancer screening includes.	Timely treatment of cervical erosion	47.2	20.3
K3. Basic methods for cervical cancer screening include?	Liquid-based thin-layer cytology test (TCT)	51.0	8.2
	B-ultrasound	68.8	
K4. High-risk factors for cervical cancer include.	Multiple pregnancies or births	42.8	3.9
	Long-term oral contraceptives	40.5	
	Long-term smoking	31.4	
K5. Diseases that HPV infection may lead to include?	Endometrial cancer	43.1	0.7
	Penile cancer	32.3	
	Oral cancer	10.5	

Multiple-choice questions will be credited with 1 point for a correct choice, and no points will be deducted for wrong decisions, omissions, or multiple options.

### Attitude toward cervical cancer screening

Four hundred and thirty-four (98.9%) participants held a positive attitude towards cervical cancer screening, believing that “every woman should undergo regular screening” (Table 3).

**Table 3:** Attitude toward cervical cancer screening among women vaccinated with HPV vaccines.

Topic	Agree *n* (%)	Disagree *n* (%)
Every woman should be screened regularly for cervical cancer.	434(98.9)	5(1.1)
I have been vaccinated against HPV and do not need to be screened for cervical cancer.	5(1.1)	434(98.9)
I thought I was healthy enough that I did not need to be screened for cervical cancer.	12(2.7)	427(97.3)
Regular cervical cancer screenings can give me peace of mind about my health.	430(97.9)	9(2.1)
I am more likely to get a cervical cancer screening if the doctor is highly skilled.	374(85.0)	65(15.0)

### Practice towards cervical cancer screening

Three hundred twenty (72.9%) had undergone cervical cancer screening after receiving the HPV vaccine, while 119 (27.1%) had not undergone screening. The main reasons for participants not being screened are shown in Table 4.

**Table 4:** Main reasons for participants not to be screened (top three).

Title	Options	*n*(%)
The main reason for not going for cervical cancer screening (*n* = 119)	Fear of the screening process	81(18.5)
	Not understanding the purpose and importance of cervical cancer screening	75(17.1)
	I maintain a healthy sex life, so I do not need cervical cancer screening	69(15.7)

### Analysis of factors influencing participants’ knowledge of cervical cancer prevention

Univariate analysis showed that there was a statistically significant difference in the knowledge scores of HPV-vaccinated women with different levels of education and occupations (combining non-medical workers into one category, which included both medical workers and non-medical workers) (*p* < 0.05) (Table 5). Including education level (“high school/secondary school and below” as the reference group) and occupation in the binary logistic regression model, the analysis showed that the Knowledge of HPV vaccines with an education level of tertiary education and above and occupation of medical workers was better (Table 6).

**Table 5:** Univariate analysis of respondents’ knowledge of cervical cancer prevention (*n* = 439).

Category	Poor knowledge	Good knowledge	$\chi^{2}$ value	*p-*value
	(*n* = 138)	(*n* = 301)		
Age			4.624	0.099
14 ~ 25	79	143		
26 ~ 35	37	111		
36 ~ 45	22	47		
Education level			22.819	<0.001
High School/Technical School and Below	24	12		
Junior College	21	50		
Bachelor’s Degree	58	143		
Master’s Degree and Above	35	96		
Marital status			2.440	0.295
Unmarried	88	184		
Married	47	115		
Divorced or Widowed	3	2		
Occupation			24.002	<0.001
Non-medical Workers	128	217		
Medical Worker	10	84		
Residence			2.174	0.537
Rural Area	31	56		
City	77	179		
Township	10	30		
County Town	20	36		
Monthly Family Income (Yuan)			3.267	0.352
<3,000	10	13		
3,001 ~ 5,000	24	40		
5,001 ~ 10,000	44	108		
>10,000	60	140		
Family History of Cervical Cancer			2.789	0.183
Yes	0	6		
No	138	295		
Received health education on cervical cancer prevention			3.391	0.066
Yes	71	183		
No	67	118		

**Table 6:** Binary logistic regression analysis of factors influencing prevention knowledge of survey respondents (*n* = 439).

Category	*B*	SE	Wald $\chi^{2}$ value	OR (95%CI)	*P-*value
Education level					
High School/Technical School and Below				Ref.	
Junior College	1.097	0.453	5.865	2.995(1.233–7.279)	0.015
Bachelor’s Degree	1.307	0.391	11.185	3.694(1.718–7.943)	0.001
Master’s Degree and Above	1.574	0.407	14.992	4.826(2.176–10.707)	<0.001
Occupation					
Non-medical Workers				Ref.	
Medical Worker	1.539	0.362	18.071	4.660(2.292–9.474)	<0.001

### Analysis of factors influencing participants’ cervical cancer screening behavior

Univariate analysis showed statistically significant differences in screening behavior between age, vaccine type, marital status, monthly household income, gynecological examination in 3 months, occupation, health education on cervical cancer prevention, and knowledge of prevention and treatment (*p* < 0.05) (Table 7). The differences between age (“14–25 years old” as the reference group), marital status (“unmarried” as the reference group), occupation, monthly family income (“<3,000 yuan” as the reference group), vaccine type (“Bivalent HPV vaccine” as the reference group), gynecological examination in 3 months, having received health education on cervical cancer prevention, and knowledge of cervical cancer prevention were included in the binary logistic regression model, and the analysis showed that women aged 26–45, married, having received health education on cervical cancer prevention and having good knowledge of cervical cancer prevention would go to be screened (Table 8).

**Table 7:** Univariate analysis of screening behavior of survey respondents (*n* = 439).

Category	*n*(%)	Screened	Not screened.	$\chi^{2}$ value	*P-*value
		(*n* = 320)	(*n* = 119)		
Age				77.176	<0.001
14 ~ 25	222(50.6)	121	101		
26 ~ 35	148(33.7)	134	14		
36 ~ 45	69(15.7)	65	4		
Education level				0.290	0.962
High School/Technical School and Below	36(8.2)	26	10		
Junior College	71(16.2)	51	20		
Bachelor’s Degree	201(45.8)	149	52		
Master’s Degree and Above	131(29.8)	94	37		
Marital status				57.909	<0.001
Unmarried	272(62.0)	164	108		
Married	162(36.9)	152	10		
Divorced or Widowed	5(1.1)	4	1		
Occupation				6.158	0.013
Non-medical Workers	345(78.6)	242	103		
Medical Worker	94(21.4)	78	16		
Residence				6.751	0.080
Rural Area	87(19.8)	60	27		
City	256(58.3)	198	58		
Township	40(9.1)	25	15		
County Town	56(12.8)	37	19		
Monthly Family Income (Yuan)				26.554	<0.001
<3,000	23(5.2)	9	14		
3,001 ~ 5,000	64(14.6)	39	25		
5,001 ~ 10,000	152(34.6)	108	44		
>10,000	200(45.6)	164	36		
Vaccine types				23.679	<0.001
Bivalent HPV vaccine	48(10.9)	45	3		
Quadrivalent HPV vaccine	86(19.6)	73	13		
Nine-valent HPV vaccine	305(69.5)	202	103		
Gynecological examination within 3 months				14.104	<0.001
Yes	98(22.3)	86	12		
No	341(77.7)	234	107		
Family History of Cervical Cancer				0.119	0.664
Yes	6(1.4)	4	2		
No	433(98.6)	316	117		
Received health education on cervical cancer prevention				7.810	0.005
Yes	254(57.9)	198	56		
No	185(42.1)	122	63		
Knowledge of cervical cancer prevention				88.133	<0.001
Poor	138(31.4)	60	78		
Good	301(68.6)	260	41		
Screening attitudes				0.425	0.412
Positive	434(98.9)	317	117		
Negative	5(1.1)	3	2		

**Table 8:** Binary logistic regression analysis of factors influencing screening behavior among survey respondents (*n* = 439).

Category	*B*	SE	Wald $\chi^{2}$ value	OR (95%CI)	*P-*value
Age					
14 ~ 25				Ref.	
26 ~ 35	2.006	0.488	16.905	7.431(2.856–19.331)	<0.001
36 ~ 45	2.439	0.824	8.756	11.466(2.279–57.694)	0.003
Marital status					
Unmarried				Ref.	
Married	1.460	0.554	6.955	4.307(1.455–12.750)	0.008
Divorced or Widowed	1.674	1.366	1.501	5.333(0.366–77.625)	0.221
Occupation					
Non-medical Workers				Ref.	
Medical Worker	−0.484	0.396	1.492	0.616(0.284–1.340)	0.222
Monthly Family Income (Yuan)					
<3,000				Ref.	
3,001 ~ 5,000	−1.057	0.564	3.516	0.348(0.115–1.049)	0.061
5,001 ~ 10,000	−0.323	0.417	0.600	0.724(0.320–1.638)	0.438
>10,000	−0.116	0.344	0.113	0.890(0.453–1.749)	0.736
Vaccine types					
Bivalent HPV vaccine				Ref.	
Quadrivalent HPV vaccine	−1.684	0.833	4.083	0.186(0.036–0.951)	0.043
Nine-valent HPV vaccine	−0.730	0.767	0.907	0.482(0.107–2.165)	0.341
Gynecological examination within 3 months					
No				Ref.	
Yes	0.140	0.466	0.090	1.150(0.461–2.869)	0.764
Received health education on cervical cancer prevention					
No				Ref.	
Yes	0.754	0.305	6.117	2.125(1.169–3.863)	0.013
Knowledge of Cervical Cancer Prevention					
Poor				Ref.	
Good	2.820	0.337	69.598	16.770(8.667–32.451)	<0.001

### Information needs

Of the 254 participants who had received health education, 34.2% had unmet needs for information on cervical cancer prevention, and 29.5% wanted professionals to provide them with health education services. 40.7% of the participants wanted to receive education services at community health service centers, followed by campuses (33.4%) and online virtual platforms (20.5%).

## Discussion

The study’s results showed that Chinese women who had been vaccinated against HPV had good overall Knowledge of cervical cancer prevention, and positive attitudes and behaviors toward cervical cancer screening but insufficient knowledge about cervical cancer screening, gaps in screening attitudes and behaviors, and unmet information needs. Age, marital status, whether or not they had received cervical cancer prevention education, and the level of prevention knowledge were the main factors influencing participants’ cervical cancer screening behavior.

The results of the study showed that community health service centers (35.0%) were the most critical source of participants’ knowledge about cervical cancer prevention, followed by campuses (26.1%) and online virtual platforms (19.8%). It can be seen that community health centers play a vital role in the dissemination of knowledge about cervical cancer prevention, which is consistent with the findings of Bo et al. (24). The fact that 19.8% of the participants in this study learned about cervical cancer prevention through online social media may be because the sample had a high literacy level and was younger, with more exposure to new media (25). In addition, among the participants who had received health education, 34.2% had unmet information needs about cervical cancer prevention, and 29.5% of the women wanted professionals to provide them with health education. This may be because the current health education content is not comprehensive, in-depth, and not targeted enough (26), and the health education is in a single form. Therefore, in the future, the role played by community health service centers in prevention should continue to be strengthened. Communities and campuses can invite professionals to educate and publicize cervical cancer prevention knowledge. Professionals can also be encouraged to produce popular science resources on cervical cancer prevention knowledge and disseminate them through new media or social media. At the same time, the relevant personnel should pay more attention to the exact needs and input of the participants when carrying out health education, and adopt diversified educational methods and materials to ensure the comprehensiveness and pertinence of the information.

The survey results showed that 68.6% of the participants had good knowledge about cervical cancer prevention. However, some of them still had misconceptions about specific knowledge about cervical cancer prevention. For example, 55.8% of the participants were not aware that HPV infection was usually asymptomatic, which might prompt them to ignore HPV infection, delay early detection and treatment, and increase the risk of cervical cancer. 31.2% of the participants believed that ultrasound was a primary method of cervical cancer screening, and 53% felt that cervical cancer screening could provide timely treatment for celiac disease. This indicates that some participants, despite having received the HPV vaccine, had a relatively limited understanding of cervical cancer screening. Consequently, they might miss out on proper screening and treatment for cervical cancer. Therefore, women who have received the HPV vaccine should remain a key focus of educational initiatives. Health educators can distribute publicity posters, educational brochures, and videos on cervical cancer prevention and treatment at all vaccination sites, utilizing both online and offline channels. Additionally, artificial intelligence technology can be harnessed to create digital health butlers for vaccinated women. These digital assistants can offer personalized screening schedules, methods, and health information based on the users’ health records and knowledge gaps. This approach could significantly reduce the incidence rate of cervical cancer in China. In addition, the results of the multifactorial analysis showed that participants with a college education and above and medical work as their occupation had better knowledge of prevention. For those with higher education and medical workers, their sources of knowledge in various aspects may be more prosperous. As confirmed in previous studies, they may pay more attention to health and disease prevention (27).

Therefore, in the future, the prevention of cervical cancer should be precise, targeting women with low literacy and non-medical backgrounds. When disseminating prevention knowledge, simple and easy-to-understand language and expressions should be used to ensure that the educational information is accessible to the intended audience (28). It is worth noting that whether or not they had received health education did not affect the respondents’ level of prevention knowledge in the univariate analysis. This may be because the study participants were not actively acquiring health education their schools or communities provided. Passive education may make it difficult to enhance their subjective awareness (26), and the learning effect is not satisfactory. In addition, some participants learned about cervical cancer prevention through online media. However, online health information of varying quality may also affect their correct knowledge about cervical cancer prevention (29). Therefore, in the future, relevant personnel should adopt interactive and participatory teaching methods to enhance participation and initiative in learning the educated to improve health education’s effect (30). Meanwhile, the government and related organizations should strengthen online health information quality supervision and guide the public in identifying reliable information sources through various means. The study results showed that 98.9% of the participants had a positive attitude toward cervical cancer screening, believing that “every woman should be screened regularly.” Related studies have also found that women with positive attitudes have higher participation rates in cervical cancer screening (31). Eighty-five percent of the women in this study indicated that they were more likely to go for cervical cancer screening if the doctor’s skill level was high. Studies have shown that the skill level of physicians directly affects the patient experience and service quality (32). Highly skilled physicians can provide a more accurate and comfortable screening process, increasing women’s willingness to participate. Therefore, cervical cancer screening technicians should focus on improving their technical level to alleviate women’s discomfort during screening, thus better-facilitating women’s cervical cancer screening.

The study showed that women with good knowledge of cervical cancer prevention would go for screening, which agrees with the report of Khumalo et al. (33). Ran et al. (34) showed that the higher the knowledge of cervical cancer prevention among the population, the higher the likelihood of realizing the importance of cervical cancer screening and the more pronounced the tendency to participate in cervical cancer screening. The results of the study showed that women between the ages of 26 and 45 years old would go for screening, similar to the results of other studies (35), probably because women between the ages of 26 and 45 years old generally already have some sexual experience and are sexually active, facing a higher risk of sexually transmitted infections. Hence, they are more motivated to participate in screening (36). Studies have shown that being married is a facilitator of cervical cancer screening in women, in line with reports by Abboud et al. (37), Mai et al. (38), and others. It may be because married people have family care and more sources of social support (39), and married people have a stronger sense of responsibility for their families and pay more attention to their health. Therefore, married people have higher adherence to cervical cancer screening than unmarried people (40). Studies have shown that participants who have been educated about cervical cancer prevention and treatment will go for screening. A systematic review (41) reported that health education positively improved attitudes toward cervical cancer screening, increased their knowledge of prevention, and enhanced their screening rate. Therefore, schools and health service organizations should conduct practical health education activities to improve women’s cervical cancer prevention knowledge. In particular, as school education in China is mostly for unmarried women under 26 years of age, it is more important to seize the opportunity of school education to disseminate knowledge about cervical cancer prevention by using health education forms that are popular among adolescents, which is also a meaningful way to increase cervical cancer screening rates and reduce the incidence of cervical cancer.

The study results showed that 98.9% of the participants had a positive attitude towards cervical cancer screening, but the % of those who engaged in screening behavior was 72.9%. This indicates a gap between participants’ screening attitudes and behaviors. According to the theory of staged behavioral change (42), knowledge and attitude belong to the pre-intentional and intentional stages, respectively, and are the basis for behavioral change. However, the influence on behavioral change may be interfered with or limited by other factors (e.g., time constraints, insufficient healthcare resources, social pressures, economic burdens, etc.). Research has shown that health behavior change is a complex process influenced by many factors. For example, a study by Thomas found that changes in adverse behaviors in high-risk individuals with type 2 diabetes were associated with a good doctor-patient relationship and effective communication (43). A study by Thomas et al. (44) suggests that factors promoting lifestyle change behaviors in pediatric patients include the clinical environment, the patient’s health beliefs, and family culture. Therefore, future studies may focus on the mechanisms of attitude-to-behavior change for cervical cancer screening to identify the critical reasons for the gap between attitude and behavior.

In conclusion, cervical cancer screening is a secondary measure of cervical cancer prevention, which occupies an important position in cervical cancer prevention and is crucial for early detection of cervical cancer. The popularization of knowledge is an essential foundation for improving the coverage and effectiveness of screening. Currently, Chinese women who have been vaccinated against HPV have positive attitudes and behaviors toward cervical cancer screening. However, their knowledge of cervical cancer prevention still needs to be improved. Their information needs are not yet met, so the relevant departments should take appropriate measures to further increase the rate of cervical cancer screening among Chinese women.

### Limitations and strengths

This study has limitations. First, it was a cross-sectional study, which prevented us from directly inferring causal relationships between participants’ knowledge, attitudes, and behaviors regarding cervical cancer prevention. Second, the survey was conducted in a community in a city in east-central China, and the convenience sampling method was used. This may have resulted in a sample that was not fully representative of Chinese women who had been vaccinated against HPV, limiting the generalizability of the results concerning screening attitudes and behaviors. Therefore, multi-stage stratified random sampling could be used in the future to obtain a more representative sample. Furthermore, because the survey adopted a participant self-report format, the respondents may have been inclined to give answers that conformed to social norms, leading to acquiescence bias. Despite these limitations, it is worth noting that this paper is the first study to focus on cervical cancer prevention and treatment knowledge, attitudes, behaviors, and information needs among Chinese women who have been vaccinated against HPV. This study may provide a reference basis for further improving the prevention and treatment of cervical cancer among Chinese women and increasing the screening rate.

## Conclusion

Our survey reported cervical cancer prevention knowledge, attitudes and behaviors, and information needs among HPV-vaccinated women in China. Education and occupation were the key factors influencing Knowledge of cervical cancer prevention and treatment. The key factors influencing their screening acceptance behavior were age, type of vaccination, marital status, whether they had received health education related to cervical cancer prevention, and good knowledge of prevention and control. In the future, efforts should be made to strengthen the popularization of science and to have universal and focused interventions through multiple ways and means to increase the screening rate of cervical cancer and promote women’s health.

## Data Availability

The data analyzed in this study is subject to the following licenses/restrictions: the datasets used and/or analyzed during the current study are available from the corresponding author on reasonable request. Requests to access these datasets should be directed to Lunfang Xie, 527548725@qq.com.
