# Supplementary material for: Knowledge, attitudes, behaviors, and information needs of women vaccinated with the HPV vaccine regarding cervical cancer prevention: a cross-sectional study
Source: Front Public Health. 2025 Feb 13;13:1493589. doi: 10.3389/fpubh.2025.1493589 (PMC11865027; doi:10.3389/fpubh.2025.1493589)
Supplement: Supplementary file 1 [file Table_1.docx]

**Questionnaire on Cervical Cancer Prevention Knowledge, Attitudes, and Behaviors Among Women Who Have Received HPV Vaccine**

Dear Women.

Greetings! Thank you for taking time out of your busy schedule to complete this questionnaire. I am a professional postgraduate student at the School of Nursing, Anhui Medical University. I sincerely hope to get your help and ask you to take a small quantity of time to fill out this questionnaire so that I can better understand the knowledge, attitude, and behaviour of HPV-vaccinated women regarding cervical cancer prevention and screening.

This questionnaire is anonymous, and the information obtained is for academic research only, so please feel free to fill it out. Thank you again for your support and cooperation!

I wish you good health and success in your work!

**Part I: Basic Information**

1. Have you been vaccinated against HPV? **[Single choice question]** *

| ○A. Yes |
| --- |
| ○B. No (Please skip to the end of the questionnaire to submit your answer) |

2. What type of vaccine did you choose? **[Single choice]** *

| ○Bivalent HPV vaccine |
| --- |
| ○Quadrivalent HPV vaccine |
| ○Nine-valent HPV vaccine |
| ○Unknown |

3. Have you had a gynaecological examination in the last 3 months? **[Single choice question]** *

| ○Yes ○No |
| --- |
|  |

4. Is there a family history of cervical cancer? **[Multiple choice question]** *

| ○Yes ○No |
| --- |

5. What is your age? **[fill in the blank]** *

_________________________________

6. What is your level of education? **[Single-choice question]** *

| ○Primary and below |
| --- |
| ○Junior High School |
| ○High school/Junior college |
| ○ College |
| ○Bachelor's degree |
| ○ Graduate students and above |

7. Your occupation? **[Single choice]** *

|  |
| --- |
| ○ students |
| ○Doctor or healthcare worker |
| ○Educator |
| ○Business or office worker |
| ○Self-employed and freelance |
| ○Enterprise/institution working |
| ○Service workers (restaurants, retail, etc.) |
| ○Retirees |
| ○Government or public sector workers |
| ○Unemployed or unemployed |
| ○Other (please specify) _________________ |

8. What is your marital status? **[Single-choice question] ***

| ○Unmarried |
| --- |
| ○Married |
| ○Divorced |
| ○Widowed **(Please skip to question 10)** |

9. Literacy of male partner. **(No male partner may be excluded.) [Multiple choice]**

| ○Primary School |
| --- |
| ○Junior High School |
| ○High school/Junior college |
| ○ College |
| ○Bachelor's degree |
| ○ Graduate students and above |

10. In which of the following ranges does your total monthly household income fall(Yuan)? **[Single-choice question]** *

| ○＜1000 |
| --- |
| ○1001～3000 |
| ○3001-5000 |
| ○5,001-10,000 |
| ○ > 10,001 |

11. Where does your family usually live? **[Single choice]** *

| ○Rural Area |
| --- |
| ○City |
| ○Township |
| ○County Town |

Part II: HPV and Cervical Cancer Knowledge

12. HPV (human papillomavirus) is not associated with cervical cancer. **[Multiple choice question] ***

| ○Right ○Wrong |
| --- |

13. HPV infection usually has no obvious symptoms. **[Multiple choice question]** *

| ○Right ○Wrong |
| --- |

14. The HPV vaccine completely prevents cervical cancer. **[Single-choice question]** *

| ○Right ○Wrong |
| --- |

15. the body's immune system clears Most HPV infections. **[Single-choice question]** *

| ○Right ○Wrong |
| --- |

16. High risk factors for cervical cancer. **[Multiple choice] ***

| Multiple sexual partners |
| --- |
| □ Early sexual intercourse |
| □ Long-term use of oral contraceptives |
| □ HPV high-risk infection |
| □ Family history of cervical cancer |
| □ High intake of vitamin C |
| □ Multiple pregnancies and multiple births |
| □ Intrauterine device |
| □ Long-term smoking |
| □ Eating chocolate for a long time |
| □ Compromised immune system |

17. Cervical cancer is predominantly found in older women; younger women do not need to worry about cervical cancer. **[Multiple choice question] ***

| ○ Correct ○ Incorrect |
| --- |

18. Persistent HPV infection may lead to cervical cancer. **[Single-choice question] ***

| ○Right ○Wrong |
| --- |

19. You cannot get cervical cancer after menopause. **[Multiple choice] ***

| ○Right ○Wrong |
| --- |

20. In your opinion, who is at risk of developing cervical cancer? **[Multiple choice] ***

| □ All ages |
| --- |
| □40-60 married women |
| □ No sexual debut |
| □ Multiple pregnancies |
| □ Multiple sexual partners |
| □ Smokers |
| □ People with a family history of cervical cancer |

21. What do you think is the main route of HPV transmission? **[Multiple choice question] ***

| ○Sexually transmitted |
| --- |
| ○Airborne |
| ○Mother-to-child transmission |
| ○Diet spread |
| ○Anything above |

22. Do you think HPV infection or having cervical cancer is related to sexual partners? **[Multiple choice question] ***

| ○Yes ○No |
| --- |

23. What diseases do you think may result from HPV infection? **[Multiple choice] ***

| □ Cervical Cancer |
| --- |
| □ Endometrial cancer |
| □ Penile cancer |
| □ Oral Cancer |
| □ Genital warts |
| □ Rectal Cancer |
| □ Ovarian Cancer |
| □ Bladder Cancer |
| □ Other (please specify) _________________ |

24. What is an effective way to prevent HPV infection? **[Multiple choice] ***

| Safe sex |
| --- |
| □ HPV vaccination |
| □ Avoid starting sex too early |
| □ Regular cervical cancer screening |
| □ Multiple pregnancies and multiple births |
| □ Quit smoking |
| □ Avoid multiple sexual partners |
| □ Other (please specify) _________________ |

**Part III: Screening knowledge**

25. Women who are regularly screened for cervical cancer will not get cervical cancer. **[Single-choice question] ***

| ○Correct |
| --- |
| ○Error |

26. Women should begin cervical cancer screening as soon as they have their first sexual encounter. **[Single-choice question] ***

| ○Right ○Wrong |
| --- |

27. Cervical cancer screening is not required for only one sexual partner. **[Single-choice question] ***

| ○Right ○Wrong |
| --- |

28. Cervical cancer screening is required if you are sexually active. **[Multiple choice] ***

| ○Right ○Wrong |
| --- |

29. Which of the following is a primary screening method for cervical cancer? **[Multiple choice question] ***

| □ Ultrasound |
| --- |
| □ Routine blood tests |
| □ Full Body Physical Examination |
| □ Liquid-based thin layer cytology (TCT) |
| □ Visual Inspection |
| □ Human papillomavirus (HPV) Testing |
| □ Gynaecology (dual clinic) |
| □ Cervical smear |
| □ Cervical biopsy |

30. In your opinion, does the purpose of cervical cancer screening include ()? **[Multiple choice] ***

| □ Early detection of cervical cancer or precancerous cervical lesions |
| --- |
| □ Provide early treatment opportunities to improve cure rates |
| □ Timely treatment of celiac disease |
| □ Reduction of neonatal mortality |
| □ Reduction of morbidity and mortality from cervical cancer |
| □ Detecting HPV infection |
| □ Improvement of women's weight or shape |
| □ Advancing medical technology |

31. There are no abnormalities that would eliminate the need for cervical cancer screening. **[Multiple choice] ***

| ○Right ○Wrong |
| --- |

32. Cervical cancer screening can affect fertility. **[Multiple choice question] ***

| ○Right ○Wrong |
| --- |

33. If you have been screened for cervical cancer before and the test results are normal, you do not need to do it again in the future. **[Multiple choice question] ***

| ○Right ○Wrong |
| --- |

**Part IV: Attitudes**

34. Every woman should be screened regularly for cervical cancer. **[Multiple choice question] ***

| ○Agree ○Disagree |
| --- |

35. I have been vaccinated against HPV and do not need to be screened for cervical cancer. **[Multiple choice question] ***

| ○Agree ○Disagree |
| --- |
|  |

36. I think I am healthy, and there is no need for cervical cancer screening. **[Multiple choice question] ***

| ○Agree ○Disagree |
| --- |

37. Regular cervical cancer screening can give me peace of mind about my health. **[Multiple choice question] ***

| ○Agree ○Disagree |
| --- |

38. Normally, there are seven days in a week. **[Multiple choice] ***

| ○Right ○Wrong |
| --- |

39. I am more likely to go for cervical cancer screening if my doctor is highly skilled. **[Multiple choice question] ***

| ○Agree ○Disagree |
| --- |

**Part V: Behavior**

40. I will go for cervical cancer screening after HPV vaccination. **[Single-choice question] ***

| ○ Yes **(Please skip to Question 41)** |
| --- |
| ○No **(Please skip to Question 42)** |

41. The reason for going for cervical cancer screening is? **[Multiple choice] ***

| □ Increased interest in information on cervical cancer prevention after vaccination |  |
| --- | --- |
| □I think cervical cancer screening is still essential . |  |
| □I am worried that the vaccine will not cover all subtypes of HPV. |  |
| □ The medical staff informed me of the need for screening during vaccination. |  |
| □ I've been positive before, and I need to be retested. |  |
| □There's a free screening program. |  |
| □I'm symptomatic. |  |
| □Routine Physical Examination |  |
| □Dr. Hutchinson recommended that |  |
| □There's a family history. |  |
| □ Other reasons (please specify) _________________ |  |

***Skip to question 43 after completing this question.**

42. Why is the main reason for not getting cervical cancer screening? **[Multiple choice question] ***

| □ Vaccinated, reduced risk of cervical cancer |
| --- |
| □ HPV vaccine can completely prevent |
| □ Not understanding the purpose and importance of cervical cancer screening |
| □ Fear of the screening process |
| □ Worry that people will gossip |
| □ No time to get screened |
| □I've never had sex. |
| □ I maintain a healthy sex life, so I don't need to be screened for cervical cancer. |
| □ No one else has done it, so I don't have to. |
| □ Worried that cervical cancer screening will harm the uterus |
| □The family disagrees. |
| □Religion |
| □Expensive |
| □ Other reasons (please specify) _________________ |

Part VI: Information needs

43. Have you ever received health education about cervical cancer prevention and treatment? **[Single choice question] ***

| ○ Yes **(Please skip to Question 44)** |
| --- |
| ○No **(Please skip to question 49)** |

44. Do you receive health education on cervical cancer, including ( )? **[Multiple choice] ***

| □ HPV vaccination age recommendation and number of doses |
| --- |
| □ What is the HPV vaccine, and what does it prevent? |
| □ Safety and side effects of HPV vaccine |
| □ How to make an appointment for HPV vaccination |
| □Cost and Insurance Coverage of HPV Vaccine |
| □ Cost and insurance coverage of cervical cancer screening |
| □ Different methods of cervical cancer screening and their advantages and disadvantages |
| □ How to make an appointment and get screened for cervical cancer |
| □ Recommendations for frequency and age of cervical cancer screening |
| □ Explanation of the cervical cancer screening process |
| □ Association between HPV and cervical cancer |
| □ Other (please specify) _________________ |

45. Where do you receive health education on cervical cancer? **[Multiple choice] ***

| □ Community Health Center |
| --- |
| □ school or college campus |
| □ Meeting room or lecture hall |
| □ Online virtual platforms (e.g., videoconferencing) |
| □Home visits |
| □Mobile sanitation vehicles |
| □Medical Office |
| □ Other (please specify) _________________ |

46. Have your information needs on cervical cancer prevention and treatment been met? **[Multiple choice question] ***

| ○ Yes **(Please skip to Question 47)** |
| --- |
| ○No **(Please skip to Question 48)** |

47. How do you get information about cervical cancer prevention and treatment? **[Multiple choice question] ***

| □ Traditional media-related channels (newspapers, TV programs, radio, etc.) |
| --- |
| □ Network social media channels (WeChat, QQ, Weibo, Xiaohongshu, etc.) |
| □ Medical-related means (medical checkups, medical visits, hospital information materials, etc.) |
| □ Discuss with friends, coworkers or family |
| □ Participate in health talks organized by the community or hospital |
| □ Other (please specify) _________________ |

***Please complete this question and end your answer.**

48. Which of the following information about cervical cancer prevention and treatment have you not yet met ()? **[Multiple choice] ***

| □ HPV vaccination age recommendation and number of doses |
| --- |
| □ What is the HPV vaccine, and what does it prevent? |
| □ Safety and side effects of HPV vaccine |
| □ How to make an appointment for HPV vaccination |
| □Cost and Insurance Coverage of HPV Vaccine |
| □ Cost and insurance coverage of cervical cancer screening |
| □ Different methods of cervical cancer screening and their advantages and disadvantages |
| □ How to make an appointment and get screened for cervical cancer |
| □ Recommendations for frequency and age of cervical cancer screening |
| □ Explanation of the cervical cancer screening process |
| □ Association between HPV and cervical cancer |
| □ Other (please specify) _________________ |

49. Do you actively seek information on cervical cancer prevention and treatment? **[Multiple choice question] ***

| ○Yes **(please skip to question 50)** |
| --- |
| ○No **(Please skip to question 52)** |

50. How do you get information about cervical cancer prevention and treatment? **[Multiple choice question] ***

| □ Traditional media-related channels (newspapers, TV programs, radio, etc.) |
| --- |
| □ Network social media channels (WeChat, QQ, Weibo, Xiaohongshu, etc.) |
| □ Medical-related means (medical checkups, medical visits, hospital information materials, etc.) |
| □ Discuss with friends, coworkers or family |
| □ Participate in health talks organized by the community or hospital |
| □ Other (please specify) _________________ |

***Skip to question 51 after completing this question.**

51. Does your initiative to seek information on cervical cancer prevention and treatment include ()? **[Multiple choice] ***

| □ HPV vaccination age recommendation and number of doses |
| --- |
| □ What is the HPV vaccine, and what does it prevent? |
| □ Safety and side effects of HPV vaccine |
| □ How to make an appointment for HPV vaccination |
| □Cost and Insurance Coverage of HPV Vaccine |
| □ Cost and insurance coverage of cervical cancer screening |
| □ Different methods of cervical cancer screening and their advantages and disadvantages |
| □ How to make an appointment and get screened for cervical cancer |
| □ Recommendations for frequency and age of cervical cancer screening |
| □ Explanation of the cervical cancer screening process |
| □ Association between HPV and cervical cancer |
| □ Other (please specify) _________________ |

52. Would you like to receive health education services from professionals? **[Single choice question] ***

| ○Yes **(Please skip to question 53)** |
| --- |
| ○No **(Please skip to the end of the questionnaire to submit your answer)** |

53. Where would you like to receive cervical cancer health education services? **[Multiple choice question] ***

| □ Community Health Center |
| --- |
| □ school or college campus |
| □ Meeting room or lecture hall |
| □ Online virtual platforms (e.g., videoconferencing) |
| □Home visits |
| □Mobile sanitation vehicles |
| □Medical Office |
| □ Other (please specify) _________________ |

54. What would you like to receive health education on cervical cancer, including ( )? **[Multiple choice] ***

| □ HPV vaccination age recommendation and number of doses |
| --- |
| □ What is the HPV vaccine, and what does it prevent? |
| □ Safety and side effects of HPV vaccine |
| □ How to make an appointment for HPV vaccination |
| □Cost and Insurance Coverage of HPV Vaccine |
| □ Cost and insurance coverage of cervical cancer screening |
| □ Different methods of cervical cancer screening and their advantages and disadvantages |
| □ How to make an appointment and get screened for cervical cancer |
| □ Recommendations for frequency and age of cervical cancer screening |
| □ Explanation of the cervical cancer screening process |
| □ Association between HPV and cervical cancer |
| □ Other (please specify) _________________ |

**Thank you for your answers, valuable time, and contribution to this study!**
